# HSP70 protects human neuroblastoma cells from apoptosis and oxidative stress induced by amyloid peptide isoAsp7-A*β*(1–42)

**DOI:** 10.1038/cddis.2015.336

**Published:** 2015-11-19

**Authors:** M M Yurinskaya, V A Mitkevich, S A Kozin, M B Evgen'ev, A A Makarov, M G Vinokurov

**Affiliations:** 1 Laboratory of Protein Conformational Polymorphism in Health and Disease, Engelhardt Institute of Molecular Biology, Russian Academy of Sciences, Moscow, Russia; 2 Laboratory of Apoptosis Regulation, Institute of Cell Biophysics, Russian Academy of Sciences, Pushchino, Moscow, Russia

*Dear Editor,*

The initial stage of Alzheimer's disease (AD) is characterized by aggregation of monomeric amyloid-*β* (A*β*) and accumulation of A*β* as insoluble amyloid plaques,^1^ which are in dynamic equilibrium with soluble oligomers of A*β*.^2^ Formation of neurotoxic oligomers most likely proceeds via a nucleation-dependent mechanism and is initiated by structurally and/or chemically modified forms of A*β*.^3^ The A*β* isoform with isomerized aspartic acid residue at position 7 (isoAsp7-A*β*(1–42)) is one of the most common in amyloid plaques. In contrast to intact A*β*, a synthetic peptide corresponding to isoAsp7-A*β*(1–42) causes cerebral amyloidosis in AD animal models^4^ and induces neuronal cell death by apoptosis.^5^ These findings point to isoAsp7-A*β*(1–42) as an agent triggering the initial step of AD – oligomerization of endogenous A*β*.^1^ Oxidative stress has a significant role in the development of AD,^6^ leading to an increase in the major stress protein HSP70. We have previously shown that recombinant human HSP70 reduces oxidative stress in innate immunity cells,^7^ prevents death of neurons and reduces amyloid plaque burden in the brain of 5xFAD mice when administered intranasally.^8^ This study examines the protective effect of recombinant human HSP70 on apoptosis and generation of reactive oxygen species (ROS) in human neuroblastoma cells, SK-N-SH, induced by isoAsp7-A*β*(1–42).

In the concentration range of 1–10 *μ*M, isoAsp7-A*β*(1–42) induced a dose-dependent increase in the proportion of apoptotic cells and production of ROS (Figures 1a and b). However, the proportion of necrotic cells in the population treated with isoAsp7-A*β*(1–42) increased by no more than 6% compared with the control. Predominant cell death by the apoptosis pathway indicates a specific effect of isoAsp7-A*β*(1–42), and the mechanism of this effect is associated with the induction of oxidative stress. Preincubation of the cells with HSP70 resulted in a significant decrease in the proportion of apoptotic cells and the level of ROS induced by isoAsp7-A*β*(1–42) (Figure 1b). We have not detected any interaction between the isoAsp7-A*β*(1–42) and HSP70 by ITC and MST (data not shown). In addition, in experiments with cells the concentration of HSP70 (30 nM) was significantly lower than that of isoAsp7-A*β*(1–42). Thus, the protective effect of HSP70 could not be attributed to the interaction with isoAsp7-A*β*(1–42).

We have investigated the impact of various inhibitors of intracellular signaling pathways that regulate apoptosis and oxidative stress on the effect of isoAsp7-A*β*(1–42). We used wortmannin, U73122, PD98059, SB203580 and SP600125 for inhibition of PI3K, PLC, ERK, p38MAPK and JNK, correspondingly.

Although isoAsp7-A*β*(1–42) increased the level of ROS and the percentage of apoptotic cells, all inhibitors reduced these parameters in SK-N-SH cells (Figure 1c). This indicates that isoAsp7-A*β*(1–42) causes a response in the cells, linked to activation of the intracellular signaling pathways mediated by JNK, ERK, PI3K, p38MAPK and PLC. Although U73122 had no effect on the HSP70 protection against isoAsp7-A*β*(1–42), SB203580 reduced the protective effect of HSP70 against apoptosis, but did not affect the level of ROS. Shutting down protein kinases JNK, ERK and PI3K completely eliminated the HSP70 protective effect against oxidative stress and apoptosis induced by isoAsp7-A*β*(1–42). These data suggest that the observed protective effects of HSP70 are due to the activity of protein kinases JNK, ERK and PI3K.

In conclusion, we have demonstrated for the first time that the protective effect of HSP70 is realized via two main mechanisms – reduction of the oxidative stress and apoptosis induced by the peptide isoAsp7-A*β*(1–42) in human neuroblastoma cells. Signaling pathways involving protein kinases JNK, ERK and PI3K have a key role in these mechanisms. It is reasonable to believe that the protective effect of HSP70 against isoAsp7-A*β*(1–42) can also be observed at the level of the whole organism, and used as an approach for the prevention of AD by utilizing recombinant HSP70.

## Figures and Tables

**Figure 1:**
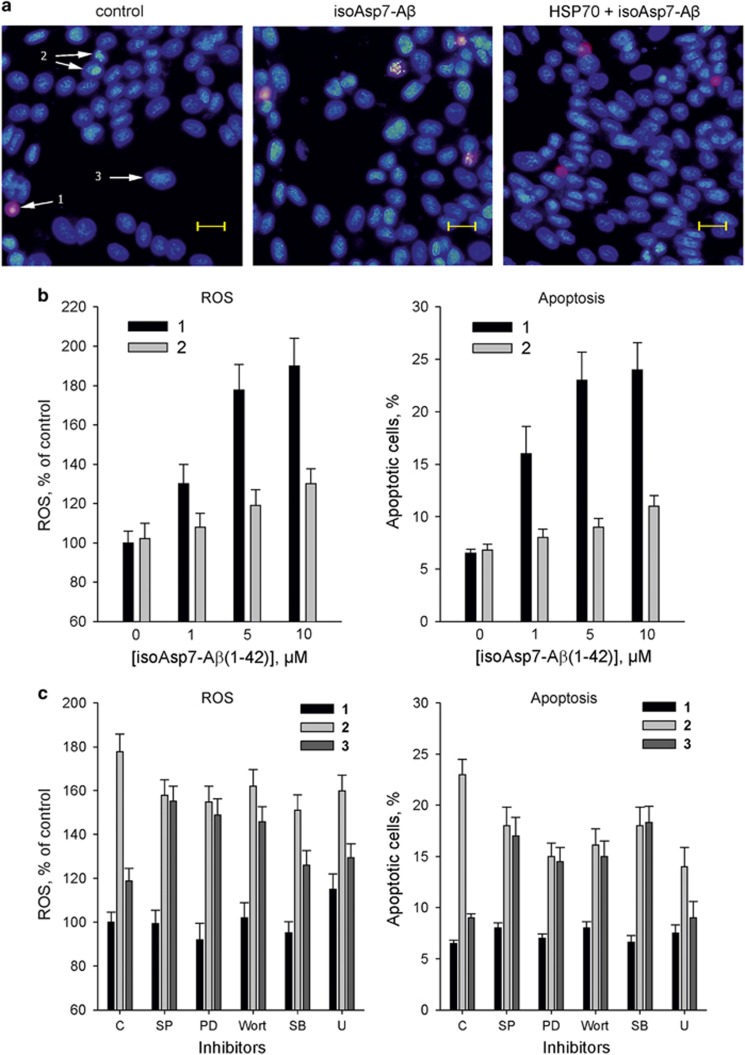
Protective effect of HSP70 against apoptosis and production of ROS induced by isoAsp7-A*β*(1–42) in neuroblastoma cells SK-N-SH. Neuroblastoma cells SK-N-SH were cultured in RPMI-1640 medium supplemented with the 10% heat-inactivated defined fetal calf serum (FCS), 2 mM l-glutamine, 100 units/ml of penicillin and 100 *μ*g/ml streptomycin at 37 °C in an atmosphere with 5% CO_2_. Before 24 h of treatment, the culture medium was replaced with the medium without FCS. Viability of the cells used in the experiments was 98–99%. Human recombinant LPS-free HSP70 expressed in armyworm (*Spodoptera*) cells was used.^7^ Synthetic peptide isoAsp7-A*β*(1–42) was purchased from Biopeptide and prepared as described in ref. 5. (**a**) Visualization of the cells using inverted fluorescence microscope Keyence BZ8100. *Left*, untreated cells. *Center*, cells treated with isoAsp7-A*β*(1–42) (5 *μ*M, 24 h). *Right*, cells preincubated with HSP70 (30 nM, 2 h), and subsequently treated with isoAsp7-A*β*(1–42) (5 *μ*M, 24 h). Scale length 20 *μ*m. Arrows indicate: 1, necrotic cells visualized by propidium iodide (PI); 2, apoptotic cells visualized by Hoechst 33342; 3, living cells. (**b**) Effect of different concentrations of isoAsp7-A*β*(1–42) and 30 nM of HSP70 on the level of ROS (left) and the amount of apoptotic cells (right) in a population of SK-N-SH cells. 1, cells treated with isoAsp7-A*β*(1–42) (24 h). 2, cells preincubated with HSP70 (30 nM, 2 h), and then treated with isoAsp7-A*β*(1–42) (24 h). The level of ROS in cells was determined by nitro blue tetrazolium: intracellular formazan was dissolved in 2 M KOH and DMSO, absorbance was measured at 620 nm. Percent of apoptotic cells was calculated as a fraction of PI-negative cells with fragmented DNA to the total number of cells (100%). To register apoptosis at least 20 fields of view were analyzed, each of which contained 250–350 cells. (**c**) The effect of inhibitors of signaling pathways on the level of ROS (left) and amount of apoptotic cells (right) in a population of SK-N-SH cells treated with HSP70 and isoAsp7-A*β*(1–42). C, control; SP, inhibitor of c-Jun N-terminal kinase (JNK), SP600125 (20 *μ*M); PD, inhibitor of extracellular signal-regulated kinase (ERK), PD 98059 (10 *μ*M); Wort, inhibitor of phosphoinositide-3-kinase (PI3K), wortmannin (100 nM); SB, inhibitor of p38 mitogen-activated protein kinase (p38MAPK), SB203580 (10 *μ*M), U, inhibitor of phospholipase C (PLC), U73122 (1 *μ*M). The inhibitors of signaling pathways were added to cells 30 min before treatment with isoAsp7-A*β*(1–42) and HSP70. 1, untreated cells; 2, cells treated with isoAsp7-A*β*(1–42) (24 h). 3, cells preincubated with HSP70 (30 nM, 2 h), and then treated with isoAsp7-A*β*(1–42) (5 *μ*M, 24 h). Each value is the mean of at least six independent experiments with triplicate samples±S.D. The comparison of data groups was performed using Student's *t*-test; *n*=6, *P*<0.005

## References

[bib1] 1Musiek ES et al. Nat Neurosci 2015; 18: 800–806. 26007213 10.1038/nn.4018 PMC4445458

[bib2] 2Cohen SI et al. Proc Natl Acad Sci USA 2013; 110: 9758–9763. 23703910 10.1073/pnas.1218402110 PMC3683769

[bib3] 3Jucker M, Walker LC. Nature 2013; 501: 45–51. 24005412 10.1038/nature12481 PMC3963807

[bib4] 4Kozin SA et al. Neurotox Res 2013; 24: 370–376. 23670398 10.1007/s12640-013-9399-y

[bib5] 5Mitkevich VA et al. Cell Death Dis 2013; 4: e939. 24287700 10.1038/cddis.2013.492 PMC3847340

[bib6] 6Radi E et al. J Alzheimers Dis 2014; 42: S125–S152. 25056458 10.3233/JAD-132738

[bib7] 7Rozhkova E et al. Ann N Y Acad Sci 2010; 1197: 94–107. 20536838 10.1111/j.1749-6632.2009.05375.x

[bib8] 8Bobkova N et al. J Alzheimers Dis 2014; 38: 425–435. 23985416 10.3233/JAD-130779

